# Receptor-Mediated Drug Delivery Systems Targeting to Glioma

**DOI:** 10.3390/nano6010003

**Published:** 2015-12-28

**Authors:** Shanshan Wang, Ying Meng, Chengyi Li, Min Qian, Rongqin Huang

**Affiliations:** Department of Pharmaceutics, School of Pharmacy, Key Laboratory of Smart Drug Delivery, Ministry of Education, Fudan University, Shanghai 201203, China; 14211030067@fudan.edu.cn (S.W.); today20090713@126.com (Y.M.); 11307120164@fudan.edu.cn (C.L.); 12307120146@fudan.edu.cn (M.Q.)

**Keywords:** glioma, blood-brain barrier (BBB), receptor-mediated, single ligand-modified targeting systems, dual ligand-modified targeting systems

## Abstract

Glioma has been considered to be the most frequent primary tumor within the central nervous system (CNS). The complexity of glioma, especially the existence of the blood-brain barrier (BBB), makes the survival and prognosis of glioma remain poor even after a standard treatment based on surgery, radiotherapy, and chemotherapy. This provides a rationale for the development of some novel therapeutic strategies. Among them, receptor-mediated drug delivery is a specific pattern taking advantage of differential expression of receptors between tumors and normal tissues. The strategy can actively transport drugs, such as small molecular drugs, gene medicines, and therapeutic proteins to glioma while minimizing adverse reactions. This review will summarize recent progress on receptor-mediated drug delivery systems targeting to glioma, and conclude the challenges and prospects of receptor-mediated glioma-targeted therapy for future applications.

## 1. Introduction

Glioma, which arises from glial cells, is by far the most frequent and lethal brain tumor, accounting for approximately 80% of malignant tumors in the central nervous systems (CNS) [1,2]. According to the growing speed and the infiltration extent to nearby brain tissues, glioma can be categorized to low-grade (World Health Organization (WHO) grades I and II), as well as high-grade (WHO grades III and IV) [3]. Even in combination of currently most effective therapies such as surgery, radiotherapy, and chemotherapy, median survival for high-grade glioma still remains gloomy (14.6 months) [4,5]. This might be attributed to specific properties of glioma, such as the highly infiltrative nature and lack of clear margins. In addition, the physical and chemical constraints of the blood-brain barrier (BBB) are also primary obstacles to the multimodality therapies for glioma [6,7,8,9].

The BBB is made up of brain capillary endothelial cells (BCECs) which are joined together by tight junctions. It plays an important role as a selective barrier and a critical regulator of brain homeostasis, separating the brain from circulating toxins and potentially harmful chemicals, and allowing access of a small subset of molecules with the appropriate size (400–600 Da), charge, and lipid solubility [8,10,11,12]. Furthermore, drug delivery to the brain is also restricted by a range of active efflux transporters present on the BBB and other contributing structures [8]. Hence, more than 98% small molecular drugs and almost all large molecules are barred from the brain tissues [13]. It has been reported that the BBB dysfunction is related to the increasing histological grade of glioma, which implies that the BBB is grossly intact at the early stage, and gradually disrupted with the progress of glioma [14]. Based on this occasion, the glioma-targeted drug delivery systems are mainly divided into two categories. When the BBB is intact, the systems which must deliver drugs across the BBB for tumor-targeting are defined as cascade-targeting systems. Other drug delivery systems utilized in high-grade glioma are designated as glioma-targeting systems, which mainly accumulated in the glioma by enhanced permeation retention (EPR) effect.

In recent years, plenty of strategies have been developed for overcoming the BBB and/or targeting to glioma such as receptor-, transporter-, or adsorption-mediated drug delivery according to different transport mechanisms [15,16]. As one of the major approaches, receptor-mediated drug delivery has been extensively studied over the past decade. This kind of drug delivery systems are comprised of nanocarriers (such as liposomes, nanoparticles (NPs), polymeric micelles, dendrimers and polymersomes), and various ligands targeting to different receptors including transferrin receptor (TfR), lactoferrin receptor (LfR), low-density lipoprotein receptor (LDLR), and folate receptor (FR) [17,18,19,20]. In this review, recent progress on receptor-mediated drug delivery systems targeting glioma is summarized according to the amount and type of the targeting moieties.

## 2. Single Ligand-Modified Targeting Systems

**Figure 1:**
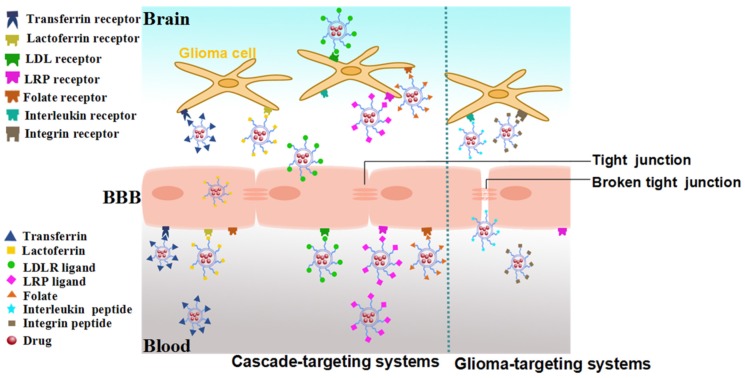
Schematic representation of single ligand-modified targeting systems.

The single ligand-modified drug delivery systems are conjugated with just one ligand that could target to both BBB and glioma, or glioma alone, which could realize cascade-targeting or glioma-targeting functions, respectively. The detailed illustration of the single ligand-modified targeting systems are shown in Figure 1 and their information are summarized in Table 1.

**Table 1:** Representative single ligand-modified targeting systems.

Target Targeting Site	BCECs	Glioma	Targeting Moiety	Nanocarrier	References
TfR	√	√	Tf	PEGylated nanoscaled GO	[21]
				Magnetic silica PLGA NPs	[17]
			OX26	Immunomicelles	[22]
			T7	Dendrigraft poly-l-lysine	[23]
				Dendrigraft poly-l-lysine	[24]
			TfR-lytic hybrid peptide	TfR-lytic hybrid peptide	[25,26]
LR	√	√	Lf	Superparamagnetic iron oxide NPs	[27]
LDLR	√	√	nLDL	nLDL	[28]
			Peptide-22	PEG-PLA NPs	[29]
LRP	√	√	Angiopep-2	Gold NPs	[30]
				PEG-PCL NPs	[31]
			MTf	**—**	[32]
FR	√	√	Folate	MnO NPs	[33]
				PEGylated PEI	[34]
Insulin Receptor	√	√	83-14 murine monoclonal antibody	PEGylated immunoliposomes	[35]
Interleukin-13 Receptor	×	√	IL-13(IP)	Mesoporous silica NPs	[36]
				Mesoporous silica-coated graphene nanosheet	[37]
Integrin	×	√	RGD	Nanochain	[38]

### 2.1. Cascade-Targeting Systems

Some receptors have been reported to be highly expressed on both BBB and glioma, which provide the cascade-targeting sites for receptor-mediated drug delivery systems. The single targeting ligand that could mediate both transportation of drugs across the BBB and their internalization into glioma cells is referred figuratively as a “multistage rocket”, which represents the simplest approach for secondary targeted delivery.

#### 2.1.1. TfR-Mediated Targeting Systems

As an essential element of the human body, cellular iron plays a crucial role in cell proliferation [39,40]. The best characterized mechanism for iron uptake is mediated by the cell surface TfR, which is classified as TfR1 and TfR2 [41,42,43,44]. Both TfR1 and TfR2 are overexpressed in some proliferating cells such as BCECs and many malignant tumor cells [21,45]. In addition, TfR is also highly expressed in some normal tissues such as liver, which limits other specific targeting efficiency *in vivo* to some extent [43]. Lots of studies have reported that TfR could be used for mediating drug delivery systems to the glioma. These systems are designed to utilize various targeting moieties such as transferrin (Tf), antibodies or specific peptides to lead carriers directly to the diseased sites.

Since TfR was found as a highlighted target for mediating drugs across the BBB, many targeting moieties aiming at TfR have been discovered. As an innate ligand, Tf is a naturally-existing protein possessing high affinity for TfR [45]. Liu and colleagues constructed Tf-conjugated doxorubicin (DOX)-encapsulated PEGylated nanoscaled graphene oxide (GO) (Tf-PEG-GO-DOX) as a BBB-targeting anti-glioma drug delivery system [21]. Quantitative determination revealed that a faster transport rate and a 4.63 intensification of cell inhibition to C6 glioma cell were achieved with the conjugation of Tf. Consistent with these results, Tf-PEG-GO-DOX also exhibited stronger inhibitory effect on tumor growth. In addition, Cui and coworkers studied the Tf-conjugated magnetic silica poly(d,l-lactic-co-glycolic acid) (PLGA) NPs loading DOX and paclitaxel (PTX) (DOX-PTX-NPs-Tf) to bypass the BBB and subsequently target to the glioma [17]. It is showed that U87 glioma cells treated by the Tf-conjugated NPs have higher intracellular accumulation of drugs than unconjugated counterparts. Additionally, the *in vivo* anti-glioma studies demonstrated that DOX-PTX-NPs-Tf treatments resulted in a substantial reduction in tumor size compared to Tf-unconjugated NPs. More recently, the use of Tf in preparation of cascade-targeting anti-glioma drug delivery systems has risen sharply, which is related to the high affinity for TfR and the effective treatments in the past studies [21,44,46]. Such conjugates with Tf have demonstrated enhanced cellular uptake via Tf-mediated mechanisms and increased selective cytotoxicity in glioma cells and tumor xenograft animal models.

However, it has been reported that high concentration of endogenous Tf (2250 nM) may produce competitive inhibition to the Tf-modified drug delivery systems [20]. Therefore, some alternatives with similar affinity for TfR were developed. Many studies have demonstrated the capacity of anti-TfR antibodies to inhibit cell growth, especially for some malignant cells [47]. For example, OX26, a mouse monoclonal antibody against rat TfR1, has been developed as a targeting moiety for delivering drugs into the brain. The first use of OX26 for the delivery of therapeutic agents across the BBB was reported in the early nineties [48]. Recently, Yue and coworkers have prepared OX26 covalently linked immunomicelles and demonstrated a two-fold increase in intracellular drug concentration with the conjugation of OX26, indicating that the OX26 modification can increase the brain uptake of micelles through TfR-mediated endocytosis [22]. Some other anti-TfR antibodies including Ri7, 8D3, and 7579 also have great potential for cascade-targeting delivery. Although these antibodies have not been verified in this field, many related achievements opened the door for Ri7, 8D3, or 7579-mediated drug delivery systems. For example, the brain distribution of Ri7 and 8D3 were characterized by Paris-Robidas *et al.* [49]. They proved that fluorolabeled anti-TfR antibodies could readily accumulated into BCECs and the antibodies were able to attach to the TfR via measuring fluorescence in brain homogenates [49]. Despite the potentials of TfR targeting antibodies for drug delivery, the fact that preparation of these antibodies is difficult within rigorous laboratory conditions makes it hard to control their quality, and limits their development in real applications.

Recently, some peptides have been greatly impressed by considerable researchers due to their facile synthesis methods and high affinity for TfR. A seven peptide (HAIYPRH, T7) has been reported to specifically bind to TfR [50,51,52]. Its high affinity for TfR is comparable to that of Tf, with the Kd of approximately 10 nM [51]. Furthermore, the T7 binding site on TfR was reported to be different from that of Tf, which might avoid interference with endogenous Tf. Interestingly, the cellular uptake of T7-conjugated drug delivery systems was found to be accelerated when endogenous Tf bound with TfR [51]. Based on this research, Jiang’s group prepared a T7-modified co-delivery system loading DOX and anti-glioma gene medicine pORF-hTRAIL (DGDPT/pORF-hTRAIL) [23]. Results showed that the T7-modified co-delivery system enhanced the cellular uptake of DOX and pORF-hTRAIL, increased their brain accumulation, and prolonged the survival time of tumor-bearing mice, compared to the system without T7 modification. Further studies demonstrated a better RNA interference effect of T7-functionalized siRNA-encapsulated NPs compared to the unmodified counterparts [24]. Consensus was reached by others, which suggesting that the T7-modified nano-enabled drug delivery systems could cross the BBB, reach the glioma and be internalized by glioma cells [53,54]. In addition to T7, another targeting peptide used to bind with TfR is TfR-lytic hybrid peptide, a combination of the TfR-binding peptide and a lytic peptide. An intriguing study designed by Kawamoto *et al.* has drawn researchers’ attention recently [25,26]. The results showed that the peptide itself was a drug delivery system which could cross the BBB and target to the glioma via the TfR-binding peptide, and further lead to cancer cell death via lysis by the lytic peptide. Cytotoxic and binding activity of the TfR-lytic hybrid peptide were verified in murine glioma GL261 cells and human glioma U251 cells, and a significant inhibited effect of tumor growth was observed in *in vivo* anti-tumor studies. As mentioned above, both the T7 and the hybrid peptide can be exploited as appropriate candidates for clinical application owing to their advantage of small steric hindrance, stability and good mediators for crossing the BBB.

#### 2.1.2. LfR-Mediated Targeting Systems

As a member of the Tf family, lactoferrin (Lf) is a single-chain cationic iron-binding glycoprotein [55]. The high concentration of endogenous Lf in human milk and its capacity to bind to the iron in breast milk suggested that Lf has a physiological function in the newborn infant [56,57]. Multiple biological activities of Lf are mediated by LfR [58]. According to some investigations on binding kinetics of Lf to target cells, different tissues or cell types seem to express their own LfR, and their characteristics vary among different cell types. Several proofs have demonstrated the presence of specific LfR in the brain [59,60]. Moreover, LfR has been proved to not only exist on the BBB in different species but also on the cell surface of glioma, which makes it a potential cascade-targeting ligand [61,62]. Compared to Tf, Lf possesses two main advantages as a brain-targeting ligand. First, the plasma concentration of Lf is much lower (approximately 5 nM), avoiding the interference of endogenous Lf [63]. Second, the transport of Lf across the BBB is unilateral, decreasing the reverse transport of Lf-modified drug delivery systems from the brain to the blood circulation [64]. A number of studies have demonstrated LfR-mediated enhancement in therapeutic effects against glioma. Su and colleagues have successfully developed Lf-modified NPs as a targeting carrier for facilitating the uptake of DOX in brain tissue and treating glioma [18].

Apart from their therapeutic benefits, these receptor-mediated systems can also play a crucial role in diagnosis of glioma. The group of Yang explored Lf-conjugated magnetic nanogels (Lf-MPNA) as a MRI contrast agent for glioma detection *in vivo* [27]. The MR images of rats’ brains after injection with Lf-MPNA nanogels displayed more significant contrast enhancement in the tumor area compared to MPNA nanogels, indicating that Lf-MPNA nanogels selectively accumulated in the tumor tissues with the help of Lf and could be used for the pre-operative MRI diagnosis of the glioma.

To sum up, Lf can be exploited as a targeting ligand conjugated to some nano-enabled carriers to yield an efficient glioma-targeting vector.

#### 2.1.3. LDLR-Mediated Targeting Systems

Low density lipoprotein receptor (LDLR), which is able to prompt the internalization of lipoprotein, has been reported to be highly expressed on the BBB and glioma cells [29,65]. Moreover, some studies on the distribution of LDLR suggested that normal brain tissues, particularly neurons, have relatively low LDLR numbers [66]. All results indicated that the LDLR is a potential molecular target for the selective delivery of anti-tumor agents to glioma [67]. Interestingly, as a natural ligand of the LDLR, LDL once was an ideal carrier for loading and delivering cancer therapeutic and diagnostic agents to targeting sites due to its structure characteristics and chemical properties. First, the size of LDL particles is precisely controlled by its apolipoprotein B-100 (ApoB-100) component through a complex network of amphipathic a-helix protein-lipid interactions [68]. Second, the native LDL is made up of 4563 amino acid residues, which serves as the binding domain for the LDLR and also keeps them soluble in aqueous environments [69]. Most of all, LDL has a highly hydrophobic core that is surrounded by a hydrophilic shell which can make LDL possess multimodal loading capabilities for embedding drugs with different affinities into the shell or the core [70]. However, many studies argued that native LDL is not suitable to act as an appropriate targeting agent because it is difficult to be isolated in large scale and is variable in composition and size. Moreover, large batches of reconstituted LDL cannot be accomplished due to the hard purification of ApoB. Thus, increasing synthetic LDL analogues are developed as replacements of native LDL. Hayavi *et al.* created a synthetic LDL particle using a lipid emulsion and a peptide composed of LDLR binding domain of ApoB [19]. This LDL particle can deliver cholesterol to cells via the LDLR. In addition, Nikanjam and coworkers developed a synthetic nano-LDL (nLDL) particle containing a lipid emulsion and a unique bifunctional peptide, which could be internalized by glioma cell lines (including SF-767 cells) [28]. After loaded with a lipophilic prodrug, paclitaxel oleate (PO), the glioma-internalizing ability was maintained and glioma cell killing effects were apparently enhanced compared to free drug. What’s more, the cell killing effect could be significantly inhibited by suramin, a LDLR inhibitor. These results suggested the LDLR-mediated mechanism of nLDL.

Some targeting moieties such as ApoB, apolipoprotein E (ApoE), or some peptides corresponding to the LDLR-binding domain of ApoE or ApoB have been proven to be effective in LDLR-targeted therapy of neoplastic diseases [71,72]. However, they are not promising candidates as targeting moieties due to some inherent drawbacks such as protein instability, competition with endogenous LDL and so on. The group of Jiang proposed that peptide-22, a peptide which showed high affinity for LDLR without competition with endogenous LDL, could be used to transport PTX-loaded NPs across the BBB and subsequently target to brain tumors [29]. The peptide-22-decorated NPs proved its targeting capacity on monolayer BBB model, glioma cells, and a BCECs/C6 glioma co-culture model *in vitro*. In the meantime, the NPs exhibited stronger brain permeation, glioma targeting, and enhanced chemotherapeutic effect compared to other groups *in vivo*. These results revealed the feasibility of peptide-22 as a cascade-targeting moiety for LDLR.

#### 2.1.4. LRP-Mediated Targeting Systems

Low density lipoprotein receptor-related protein (LRP) is a type of transmembrane proteins belonging to the LDL receptor family [73,74]. The ubiquitous LRP has been detected up-regulated in numerous human diseases, including glioma [75,76,77]. It has been reported that LRP is not only overexpressed on glioma cancerous cells but also on the BBB [31]. Moreover, LRP can serve as a common receptor of multiple ligands across the BBB such as melanotransferrin (MTf), Lf, and receptor-associated protein [32,78]. These findings confirmed the role of LRP as an ideal target that can be used for direct delivery of anti-tumor agents to tumor cells. Angiopep-2 peptide, the most famous ligand of LRP, was derived from the common peptidic sequence of the LRP protein ligands, and has shown transcytosis capacity in an *in vitro* BBB model and the ability to cross the BBB *in vivo* via the LRP-mediated pathway [79,80]. In recent years, more and more nano vehicles have been fabricated with angiopep-2 to target therapeutic agents to cross the BBB and accumulate in glioma. Ruan and colleagues developed angiopep-2-conjugated DOX-loaded gold NPs to target both BBB and glioma cells [30]. The enhanced cellular uptake and anti-glioma activity of the gold NPs are achieved because angiopep-2 could mediate glioma targeting by specific interaction with LRP. At even earlier time, Fang’s group used angiopep-2 to modify PTX-loading poly(ethylene glycol)-co-poly(ε-caprolactone) (PEG-PCL) NPs [31]. The conjugation of angiopep-2 mediated higher inhibitory effect to U87 MG glioma spheroid and larger amount of NPs in the solid glioma, demonstrating the transportation ability of angiopep-2 across the BBB and enrichment of NPs in glioma selectively. The most exciting thing is that a paclitaxel-angiopep-2 conjugate, GRN1005, has shown safe and fairly tolerant in Phase I clinical trials [81]. Although the preliminary efficacy of GRN1005 in patients with recurrent glioma is modest, the success of brain penetration increased the probability for further clinical trials. MTf, also known as a human melanoma-associated antigen p97, is a homologue of Tf [82]. It is identified as another potential targeting moiety to LRP due to its high affinity. In addition, the specific structure and functions of MTf suggest that it might be an ideal vehicle to transport drug conjugates into the brain [83]. Gabathuler *et al.* reported that the MTf-adriamycin conjugate significantly increased the survival of mice implanted with C6 glioma cells compared to injections of free adriamycin [32]. In addition, the brain’s uptake of conjugate was over eight-fold higher than that of bovine serum albumin (BSA) or Lf. Taken together, p97 may have the potential to deliver therapeutic drugs to the glioma as a viable effective carrier.

#### 2.1.5. FR-Mediated Targeting Systems

Folate is a low-molecular-weight pterin-based vitamin. FR is essential to capture exogenous folate and various derivatives into the cell cytosol due to the lack of key enzymes of the folate biosynthetic pathway in animal cells [33]. Studies demonstrated that FR is significantly overexpressed on both the BBB and glioma cells, but is sub-expressed in normal brain tissues [34]. These intriguing studies inspired researchers to develop folate-configured nano-enabled carriers as solid tumor targeting vehicles. Cheng and colleagues studied the combined DOX and BCL-2 siRNA therapy using the FR-targeted multifunctional nanopaticle for penetrating the BBB and targeting glioma [84]. The results indicated that FA-conjugated multifunctional nanocarrier could effectively deliver DOX and siRNA into C6 cells *in vitro*. *In vivo* experiments showed significant anti-glioma effect and improvement in the survival time of tumor-bearing mice when compared with other formulations, like non-targeting nanocarriers. Moreover, Chen *et al.* fabricated FA-conjugated *N*-(trimethoxysilylpropyl) ethylene diamine triacetic acid (TETT) modified MnO NPs (MnO-TETT-FA) as tumor-specific MRI contrast agents for glioma detection [34]. MR images showed the accumulation of MnO-TETT-FA NPs was more than that of MnO-TETT NPs in the brain of giloma-bearing mice. This additionally illustrated the role of FA in effective drug delivery to the glioma. Additionally, increasing studies give high priority to FR-targeting systems as possible therapeutics for glioma [85,86].

#### 2.1.6. Other Receptor-Mediated Targeting Systems

Beyond the receptors listed above, there are still several receptors being referred as targeting sites for therapeutic agents highly expressed on the BCECs and glioma. One example is the insulin receptor, a membrane glycoprotein present in BCECs as well as glioma cells [35]. Zhang *et al.* reported that PEGylated immunoliposomes (PILs) modified with 83–14 murine monoclonal antibody, an antibody to the human insulin receptor, could target the gene formulation to brain tumors *in vivo* [87]. Many studies suggested that humanized 83–14 murine monoclonal antibody had the potential to be used in clinical treatments. Another potential targeting site is the membrane-bound precursor of heparin-binding epidermal growth factor (HB-EGF), which is expressed on both BBB and neuroglial cells and also known as the diphtheria toxin receptor (DTR) [88]. Gaillard *et al.* utilized a non-toxic mutant of diphtheria toxin (known as CRM197) as the receptor-specific carrier protein which conjugated directly to horseradish peroxidase [89]. Results demonstrated that the DTR seemed to be a human applicable, safe, and effective uptake receptor which could mediate drugs targeting to the brain.

### 2.2. Glioma-Targeting Systems

Usually, with the aggravation of glioma, the proliferation and invasion of cancer cells will cause a local disruption of the BBB and produce various mediators which can increase the permeability of the capillary endothelium [14,90]. On this occasion, BBB is no longer the main barrier for the drug delivery and some specific receptors on glioma and/or neovasculatures will play crucial roles.

#### 2.2.1. ILR-Mediated Targeting Systems

It has been reported that some interleukin receptors (ILRs) are highly expressed in many tumorous cells because they can contribute to malignant progression and apoptosis resistance of various cancer types, including glioma [91]. Among these ILRs, interleukin-13 receptor (IL-13R) has already been used as a targeting site for mediating nano-enabled delivery systems to glioma.

**Figure 2:**
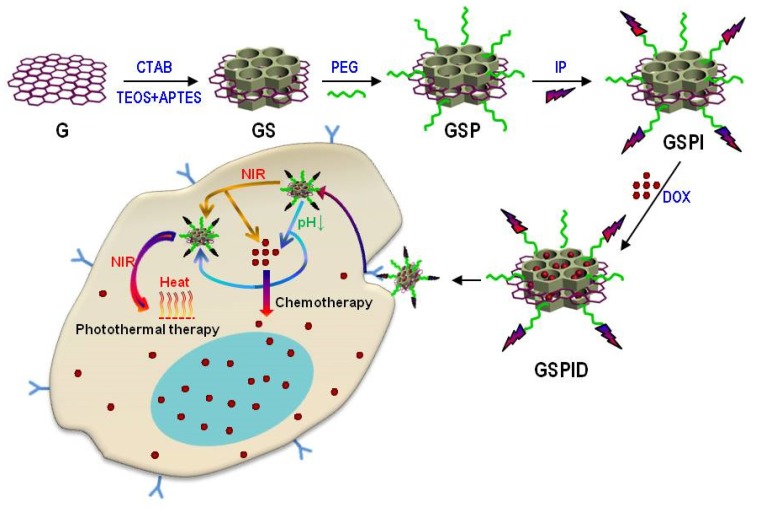
Design of the DOX-loaded GSPI-based system as a multifunctional drug delivery system for combined chemo-photothermal targeted glioma therapy.

As one of the inflammatory cytokines, interleukin 13 (IL-13) can bind to two receptor chains, IL-13Rα1 and IL-13Rα2 [92,93]. IL-13Rα2 is reported to be overexpressed on human tumors including glioma cell lines and with a high affinity for IL-13, which makes IL-13Rα2 an attractive target [92,94]. In recent years, a few attempts have been reported to utilize IL-13 to enhance therapeutic effect of glioma [36]. However, as a targeting ligand, several disadvantages, like large molecular weight and easy denaturation, still remain the major challenges of IL-13 [37]. As replacements, some peptides have been developed as targeting ligands for IL-13Rα2. IL-13 peptide (IP), which is consistent with the residues within IL-13 protein, has been demonstrated to possess high affinity for IL-13Rα2. Huang’s group developed IP-modified mesoporous silica NPs (MSN-PEG-IP) as a novel vehicle for delivering DOX to glioma cell lines for the first time [37]. The cellular uptake of MSN-PEG-IP/DOX in U251 glioma cells was significantly higher than that of unmodified NPs, indicating that IP is a potential ligand for fabricating glioma-targeting drug delivery systems. In order to further verify the targeting ability of IP, Huang and colleagues synthesized IP-modified mesoporous silica-coated graphene nanosheets (GSPI) to load DOX for combined therapy of glioma (Figure 2) [54,95]. The novel multifunctional drug delivery system showed higher cellular uptake and cytotoxicity against glioma cells than those of unmodified counterparts. All these results cemented the fact that IP is promising to target nano-enabled theranostic systems to glioma. In addition to IP, other peptides such as IL-13p and Pep-1, have also shown great targeting ability to glioma [91,96].

#### 2.2.2. Integrin-Mediated Targeting Systems

As known, integrin is a member of cell adhesion receptors’ family, playing an important role in tumor growth, invasion, and metastasis [97]. It is composed of α and β subunits. In mammals, 18 α subunits and eight β subunits assemble into 24 integrin receptors [98]. Several integrins have been reported to be highly expressed on the surface of glioma cells and the tumor neovasculatures, but minimally expressed on epithelial and mature endothelial cells [89]. These integrins support some docking sites for drug delivery systems to target to the glioma. Among these receptors, αvβ3, αvβ5, and α5β1 can specifically recognize an RGD motif, which is an exogenous peptide ligand composed of an Arg-Gly-Asp amino acid sequence [99]. In the past several years, RGD and its analogs have been widely investigated for glioma targeting as integrin ligands. Slegerova and coworkers prepared cyclic RGD-modified fluorescent nanodiamonds for targeted drug delivery to glioblastoma cells [38]. Results showed that the fluorescent nanodiamonds possessed high internalization efficacy into U87 cells with conjugation of a cyclic RGD peptide. Furthermore, Peiris *et al.* used a cyclic RGD peptide to conjugate the chain-like NPs (nanochain) [100]. After being loaded with DOX, the nanochain exhibited a 2.6-fold higher deposition in brain tumors than non-targeted counterpart, illustrating the targeting ability of the cyclic RGD peptide to glioma and the therapeutic efficacy of the nanochain treatment. Therefore, RGD has been identified as a potential targeting ligand for glioma.

## 3. Dual Ligand-Modified Targeting Systems

Two ligands in this kind of systems may play independent functions to different targeting sites, which then mediate drug delivery systems to overcome different barriers for final glioma targeting.

### 3.1. Cascade-Targeting Systems

The first strategy of the cascade-targeting systems is to modify the nanocarriers with two kinds of ligands. One of the ligand targets across the BBB, and the other ligand targets tumor cells. Gao *et al.* came up with a cascade-targeting strategy in order to achieve high and precise brain glioma targeting. They employed PEG-PCL NPs modified with two kinds of targeting ligands [101]. The cellular uptake results suggested that the first stage targeting ligand only targeted to endothelial cells which are the major cellular structure of the BBB, and the second stage ligand only delivered NPs to neuroglial cells that are involved in the majority of CNS diseases including glioma. Another study of them further indicated that this strategy could increase tumor accumulation and decrease the normal brain distribution [102].

Additionally, the second strategy of the cascade-targeting systems is to build nanocarriers modified with two kinds of ligands, with one targeting to the BBB and tumor cells, and the other promoting the targeting effect. The former ligands are usually some targeting moieties binding to their corresponding receptors, and the latter ligands may be some cell penetrating peptides or some targeting moieties binding to the corresponding receptors, transporters, and so on. Drug delivery system, G4-DOX-PEG-Tf-TAM, was synthesized with Tf and Tamoxifen (TAM) conjugatd to polyamidoamine (PAMAM) dendrimer by Li *et al.* [103]. In this system, Tf was conjugated to mediate the dual-targeting carrier to transport across the BBB and enhance its internalization in glioma. Another targeting moiety, TAM, is an estrogen receptor modulator which was used to improve the BBB permeability. The uptake ability of C6 glioma cells followed the order of G4-DOX-PEG-Tf-TAM > G4-DOX-PEG-Tf > G4-DOX-PEG, indicating that the dual ligand-modified carrier mediated the highest cellular uptake than single ligand-modified or non-targeting carriers. Further study verified the strongest transport efficiency of G4-DOX-PEG-Tf-TAM (6.06%) across the *in vitro* BBB model compared to that of G4-DOX-PEG-Tf (4.93%) and G4-DOX-PEG (4.62%). He and coworkers developed another dual ligand-mediated cascade-targeting DOX-loaded NPs conjugated with Tf and wheat germ agglutinin (WGA), which has the potential to transfer drugs across the BBB and further target glioma cells [104]. The PAMAM-PEG-WGA-Tf-DOX showed great inhibitory effect to C6 cells after crossing the BBB, compared to PAMAM-PEG-Tf-DOX, PAMAM-PEG-WGA-DOX, and free DOX, indicating better effect by treatments with dual ligand-modified systems. Based on their work, Chen and coworkers prepared polymersomes conjugated with des-octanoyl ghrelin and folate as novel BBB-penetrating and cancer-targeting delivery system [20]. As a 28 residue peptide hormone, des-octanoyl ghrelin was identified as an endogenous ligand of the growth hormone secretagogue receptor, which was observed to transport only in the blood-to-brain direction. Uptake of drugs by the C6 glioma cells through the *in vitro* BBB model and glioma in *in vivo* experiments showed that this bifunctional polymersome could enhance BBB transport and tumor accumulation. In addition, a mass of dual cascade-targeting drug delivery systems including daunorubicin-loaded liposome conjugated with p-aminophenyl-α-d-manno-pyranoside (MAN) and Tf, liposome co-modified with T7 and TAT, have been proved to possess stronger BBB-crossing and glioma-accumulating capability compared with single ligand-modified systems [98,105]. Some representative dual targeting systems are summarized in Table 2.

**Table 2:** Representative dual ligand-modified targeting systems.

Nanocarrier	Targeting Moiety	Targeting Site	Payload	Reference
PAMAM dendrimer	Tf	TfR	DOX	[104]
	WGA	WGA receptor		
PAMAM dendrimer	Tf	TfR	DOX	[103]
	TAM	ABC transporters		
PEG-PLA NPs	Lf	LfR	PTX	[106]
	tLyP-1	Neuropilin-1		
Liposomes	Angiopep-2	LRP	VEGF siRNA, Docetaxel	[107]
	tLyP-1	Neuropilin-1		
Liposomes	Tf	TfR	DOX	[108]
	TAT	—		
PEG-PCL NPs	Angiopep-2	LRP	DOX	[102]
	EGFP-EGF1	Specific tissue factor		
Polymer-lipid hybrid NPs	Folate	FR	PTX	[109]
	RGD	Integrin		
PEGylated Liposomes	OX26	TfR	PC27	[110]
	CTX	Matrix metalloproteinase-2		

### 3.2. Glioma-Targeting Systems

Similar to single ligand-modified glioma-targeting systems, the dual ligand-conjugated systems can specifically target the receptors on the glioma and/or neovasculatures. Gao *et al*. utilized RGD and IP as ligands to modify polymeric NPs (IRNPs) [111]. The modification of IP and RGD could increase the uptake amount within C6 cells through corresponding receptors and the *in vivo* study revealed that the fluorescent intensity of DiR-loaded IRNPs in glioma was 3.82-fold that of unmodified NPs. These results demonstrated that the co-modification of RGD and IP could increase the glioma targeting effect, which proved the superiority of dual ligand-modified targeting delivery systems. Although lots of dual ligand-modified targeting systems showed more satisfactory targeting effects, the systems with a single ligand are also other choices which could simplify the preparation of therapeutics. Thus, different research may choose the best way in accordance with requirements in the further.

## 4. Conclusions

For glioma diagnosis and therapy, a growing number of strategies have been and are being proposed. Compared with other strategies, receptor-mediated drug delivery is most extensively studied lying on several advantages. These systems possess a vital component: nanocarriers modified with targeting moieties. Until now, lots of targeting moieties which are ligands of the receptors on the BBB or glioma have been exploited for BBB-crossing and/or glioma-targeting drug delivery, such as potential peptide ligands. In addition, more and more research has proven that BBB/glioma-specific targeting nanocarrier can help drugs selectively target to the glioma, thus providing higher therapeutic efficiency while simultaneously decreasing systemic toxicity. It seems that the strategy has developed quite well, but so far all the systems except GRN1005 were just used for preclinical studies and none of them have completed Phase II clinical trials. This reminds us that the strategy is still a long way off. It is particularly gratifying that increasing receptors are being found on the BBB or glioma cells, which provide possible targeting sites for developing new receptor-mediated targeting systems. Therefore, extensive research is still required to identify new receptors and corresponding ligands. Most importantly, great efforts should be made to explore and facilitate potential delivery systems for glioma theranosis in future clinical applications.
